# Imaging of elbow entrapment neuropathies

**DOI:** 10.1186/s13244-025-01901-1

**Published:** 2025-01-29

**Authors:** Domenico Albano, Gabriella Di Rocco, Salvatore Gitto, Francesca Serpi, Stefano Fusco, Paolo Vitali, Massimo Galia, Carmelo Messina, Luca Maria Sconfienza

**Affiliations:** 1 IRCCS Istituto Ortopedico Galeazzi, Milan, Italy; 2 Dipartimento di Scienze Biomediche, Chirurgiche ed Odontoiatriche, Università Degli Studi di Milano, Milano, Italy; 3 Scuola di Specializzazione in Radiodiagnostica, Università Degli Studi di Milano, Milano, Italy; 4 Department of Biomedical Sciences for Health, Università Degli Studi di Milano, Milan, Italy; 5 Section of Radiology, Department of Biomedicine, Neuroscience and Advanced Diagnostics (BiND), University Hospital “Paolo Giaccone”, Palermo, Italy; 6 UOC Radiodiagnostica, ASST Centro Specialistico Ortopedico Traumatologico Gaetano Pini-CTO, Milan, Italy

**Keywords:** Nerve compression syndromes, Elbow, Ultrasonography, Magnetic resonance imaging.

## Abstract

**Abstract:**

Entrapment neuropathies at the elbow are common in clinical practice and require an accurate diagnosis for effective management. Understanding the imaging characteristics of these conditions is essential for confirming diagnoses and identifying underlying causes. Ultrasound serves as the primary imaging modality for evaluating nerve structure and movement, while MRI is superior for detecting muscle denervation. Plain radiography and CT play a minor role and can be used for the evaluation of bony structures and calcifications/ossifications. Comprehensive knowledge of anatomical landmarks, nerve pathways, and compression sites is crucial for clinicians to accurately interpret imaging and guide appropriate treatment strategies for entrapments of ulnar, median, and radial nerves, and their branches.

**Critical relevance statement:**

Accurate imaging and anatomical knowledge are essential for diagnosing elbow entrapment neuropathies. Ultrasound is the preferred modality for assessing nerve structure and motion, while MRI excels in detecting muscle denervation and guiding effective management of ulnar, median, and radial nerve entrapments.

**Key Points:**

- Ultrasound is the primary modality for assessing nerve structure and stability.
- Findings include nerve structural loss, isoechogenicity, thickening, and hyper-vascularization.
- MRI provides a comprehensive evaluation of the elbow and accurate muscle assessment.
- Imaging allows the identification of compressive causes, including anatomical variants, masses, or osseous anomalies.
- Awareness of anatomical landmarks, nerve pathways, and compression sites is essential.

**Graphical Abstract:**

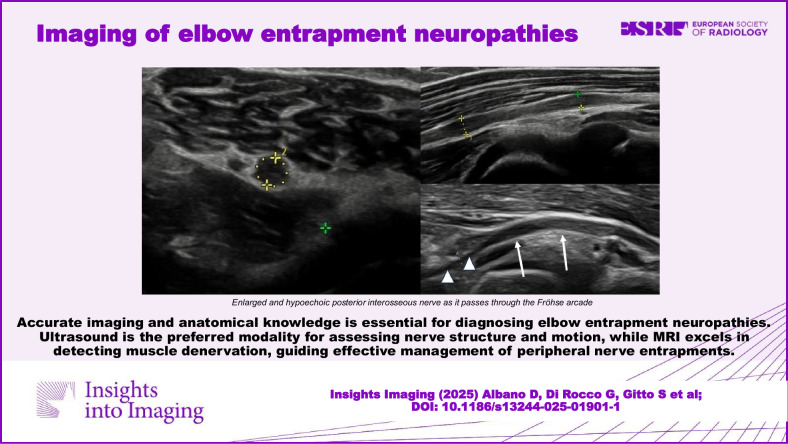

## Introduction

Entrapment neuropathies are common conditions in which peripheral nerves are compressed or stretched because of congenital or acquired disorders (or a combination of both) [1]. Diagnosis and management of these entities are based on patient history, physical and electrophysiological findings, and imaging evaluation [2]. Imaging is useful to confirm the diagnosis, determine the site of compression, identify the underlying cause, and provide an anatomical roadmap of the nerve before surgery or interventional procedures. The elbow is the crossing point of the three main nerves of the upper limb, namely the ulnar nerve (UN), radial nerve (RN), and median nerve (MN). Because of their anatomic location and the dynamic forces at the elbow, it represents a common site of compressive neuropathy. In this setting, ultrasonography (US) and magnetic resonance imaging (MRI) are the imaging modalities of choice [3, 4]; plain radiography and computed tomography (CT) have a marginal role, being helpful for the evaluation of bony deformities and soft tissue calcifications/ossifications [5].

In this narrative review, we will discuss the imaging findings of entrapment neuropathies involving the UN, MN, and RN at the elbow.

## Imaging aspects and normal appearance of peripheral nerves

### Ultrasound

It represents the main imaging modality in the evaluation of superficial peripheral nerves since it offers several benefits [6]. It is cheap and fast to perform, and it allows the evaluation of an entire nerve and comparison with the contralateral side. Moreover, US enables real-time dynamic assessment of nerve changes during flexion, extension, pronation, and supination of the elbow. US is more accurate than MRI for the evaluation of peripheral nerve thickness and structure. High-resolution linear probes are needed to assess peripheral nerves: general-purpose linear probes (up to 15 MHz) can be used to assess the main branches, although for smaller nerves, dedicated probes up to 24 Mhz and beyond should be preferred [3]. Normal nerves have a honeycomb appearance in the transverse section, where the hypoechoic nerve fascicles are surrounded by hyperechoic collagen and connective tissue, which includes endoneurium and perineurium. The margins of the nerve also appear echogenic due to the presence of epineurium. When compared to the adjacent structures, nerves are more echogenic than muscles and less sensitive than tendons to anisotropy. In the long axis, peripheral nerves have a fascicular echotexture. They appear coarse and hypoechoic and can be easily distinguished from adjacent tendons, which appear more echogenic, compact, and fibrillar [7, 8].

### Magnetic resonance imaging

**Fig. 1:**
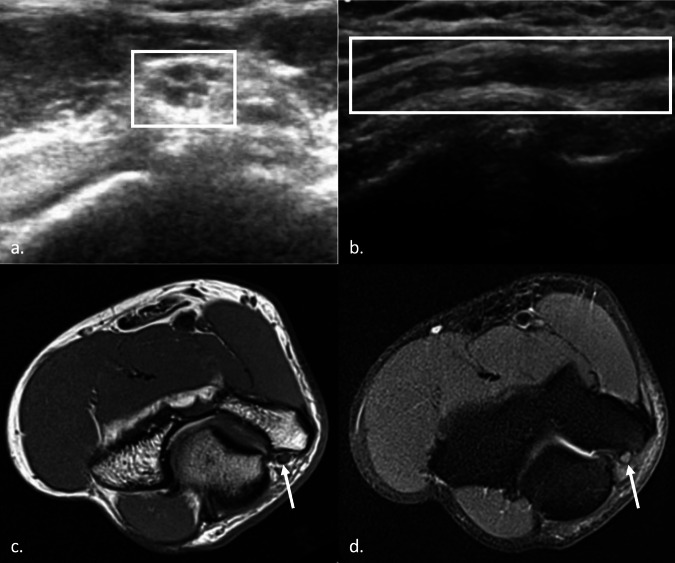
Normal nerve appearance on US and MRI examination. Transverse axis US image (**a**) and longitudinal axis US image (**b**) of UN at the level of the elbow joint. The nerve has a honeycomb appearance in the short axis (**a**) and a coarse hypoechoic appearance in the long axis (**b**). T1 (**c**) and fat-suppressed PD (**d**) MRI images of UN (arrow) in the cubital canal. The nerve appears isointense to muscle in **c** and mildly hyperintense in **d**

With its high contrast resolution, MRI enables accurate visualization of the peripheral nerves and the surrounding structures. MRI provides a more comprehensive and objective evaluation of the bone and soft tissues, presenting higher sensitivity for muscle changes related to denervation when compared to the US. At the authors’ institution, images are acquired with 1.5-T and 3-T magnets and a surface coil; the standard sequences are axial and coronal T1-weighted, axial and sagittal T2-weighted, axial and coronal fat-suppressed proton density-weighted images. A normal nerve on axial T1-weighted images appears as a smooth round or ovoid structure with a signal isointense to adjacent muscle. A (fatty) rim of hyperintense signal often surrounds peripheral nerves. On fat-suppressed proton density and T2-weighted images, nerves are mildly hyperintense compared with the signal intensity of normal muscles. Nerve fascicles may have a signal intensity slightly higher than that of the perineurium and internal perineural tissue [9, 10] (Fig. 1).

## General US and MRI features of compressive neuropathy

In the US, a pathological nerve segment will show loss of the normal honeycomb structure, decreased echogenicity, focal enlargement or neuroma formation, and, in some cases, increased vascularity on Power/Color-Doppler evaluation. Surrounding compressive masses, ganglia, accessory muscles, loose bodies, or osteophytes can be identified, although no clear compression or nerve changes can be seen in some entrapment neuropathies [11, 12]. US is routinely used for diagnosis but also as a guide for interventional procedures like perineural injections and release [13–17].

**Fig. 2:**
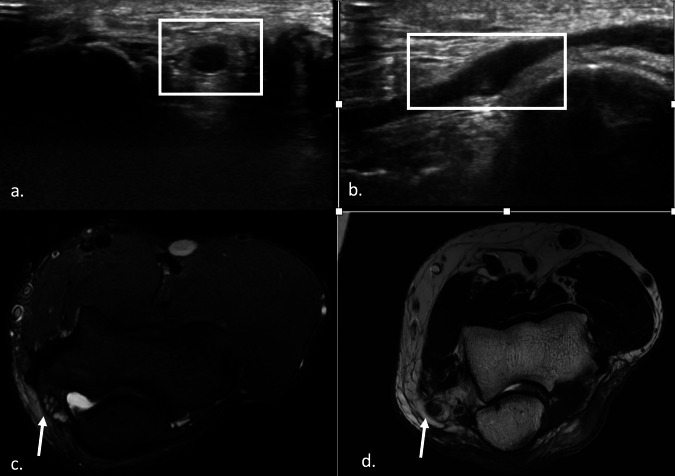
US and MRI features of ulnar compressive neuropathy. Transverse axis US image (**a**) and longitudinal axis US image (**b**) of UN at the elbow, showing focal nerve enlargement, loss of the normal honeycomb structure, and decreased echogenicity. Axial fat-suppressed PD (**c**) and T1-weighted (**d**) images of UN (arrow) in the cubital canal, showing enlarged UN with hyperintense fascicles (**c**) and inhomogeneity of the surrounding fat (**c**, **d**). In **d**, the UN is also dislocated anteriorly and medially

MRI signs of neuropathy are focal or fusiform nerve enlargement, hyperintensity, and distortion of the fascicular pattern. However, compression may alter nerve physiology without affecting its anatomy, especially in the earlier phases of the disease; in these cases, MRI may demonstrate signs of muscle involvement, which appear as a high signal intensity “edema” on fat-suppressed proton density (PD) and T2-weighted images. In advanced chronic damage, muscle atrophy and fatty changes with high signal intensity on T1-weighted can be observed [18–20] (Fig. 2). Table 1 resumes the main pros and cons of US and MRI.

**Table 1:** Main pros and cons of US and MRI

	US	MRI
Spatial resolution	+	–
Entire length	++	+
Comparative	++	+
Dynamic assessment	+	–
Fast and cheap	+	–
Contraindication	–	+
Reproducibility	+	++
Deeper nerves	+	++
Panoramic view (bones, soft tissue)	–	+
Denervation muscle changes	+	++

*US* ultrasound, *MRI* magnetic resonance imaging, *+* pros, *–* cons

## Ulnar nerve

The UN originates from the medial cord of the brachial plexus (C8, T1) with occasional contributions from the C7 nerve root. It travels along the posteromedial compartment of the upper arm, positioned medial to the axillary and brachial arteries until the mid-arm. Here, the nerve penetrates the medial intermuscular septum, entering the posterior compartment and lying anterior and medial to the triceps muscle [21]. Approximately 4–6 cm proximal to the medial epicondyle, the nerve may pass through the arcade of Struthers, a fibrous tunnel present in most individuals, formed by the medial intermuscular septum, the internal brachial ligament (a structure in the medial aspect of the posterior compartment), deep fascia, and muscle fibers of the medial head of the triceps [22].

The UN passes posterior to the medial epicondyle of the humerus in the cubital tunnel, a fibro-osseous space bound by the olecranon process laterally, the posterior cortex of the medial epicondyle medially, the elbow joint capsule and posterior bundle of the medial collateral ligament anteriorly, and the ligament of Osborne (the cubital retinaculum) posteriorly.

Exiting the distal cubital tunnel, the nerve enters the medial forearm between the heads of the flexor carpi ulnaris muscle. It continues between the superficial and deep compartments of the forearm along its medial side [23]. At the wrist, it passes through the Guyon canal, a fibro-osseous tunnel localized at the ulnar side of the palmar aspect of the wrist, and bifurcates into superficial (sensory) and deep (motor) branches [24, 25]. The UN innervates the skin and muscles of the ulnar side of the forearm and hand, including abductor digiti minimi, flexor digiti minimi brevis, opponens digiti minimi, deep head of flexor pollicis brevis, adductor pollicis, and the two lumbrical muscles on the ulnar aspect and the palmar/dorsal interossei muscles [26].

For US examination of the UN within the cubital tunnel, the transducer is placed transversely between the medial epicondyle and the olecranon. Different patient positions have been used for examining the UN. Dynamic imaging of the cubital tunnel is performed either with the patient seated and the elbow placed on a stiff pillow or, at least for the right side, with the patient supine and the arm abducted, hanging out of the table. When performing this maneuver, it is crucial to maintain a very light contact between the US probe and the patient’s skin, also with the abundant use of contact gel, as external compression may prevent the nerve from dislocating. The UN should be initially assessed with the elbow in extension and the shoulder in slight external rotation. After that, dynamic assessment of the UN can be performed by passively flexing the patient’s elbow. Videos 01–03 show how to scan the UN from the upper arm to the cubital tunnel (Video 01), how to check UN stability (Video 02), and how to follow the UN from the elbow to the forearm (Video 03). The normal mean cross-sectional area (CSA) has been reported to range between 5.6 and 6.8 mm^2^ [27, 28]. In the case of neuropathy, the US can show an increase in the CSA of the UN, measured at the level of the medial epicondyle immediately proximal to the ulnar tunnel, which is considered the best indicator of UN distress with a recommended cut-off value of 10–10.5 mm^2^ [12, 29–31].

### Cubital tunnel syndrome

Cubital tunnel syndrome is the second most common peripheral neuropathy of the upper extremity after carpal tunnel syndrome. It is the result of pathologic compression or a lesion of the UN within the cubital tunnel [32]. Ulnar neuropathy at the cubital tunnel can occur due to dynamic compression during elbow flexion movements or can result from external compression or impingement. When the elbow is flexed, the space between the humerus’s medial epicondyle and the olecranon widens, putting strain on the cubital tunnel retinaculum and thereby decreasing the ulnar canal’s diameter. Individuals at high risk of developing ulnar neuropathy include baseball pitchers, truck drivers, and office workers whose jobs involve repetitive or prolonged elbow flexion [33–35]. Extrinsic causes of compression can be masses (schwannomas or ganglia), accessory muscles (e.g., anconeus epitrochlearis muscle), elbow arthritis, bone abnormalities (due to previous fractures or surgery, osteophytes) (Video 04) [36, 37].

Common symptoms of UN compression are paresthesia and dysesthesia of the last two fingers, especially at night and during elbow flexion and repeated flexion–extension movements. The patient may also feel elbow pain that radiates towards the hand. In more advanced cases, the Wartenberg sign (the abduction stance of the small finger) can be seen, and there may be a claw deformity of the hand with extension of the metacarpophalangeal joints with flexion of the proximal interphalangeal joints, secondary to paralysis of lumbrical and interosseous muscles. Physical examination can also demonstrate a positive Tinel sign, defined as the “pins and needle feeling” elicited by tapping on the nerve proximally, with resulting paresthesia experienced in the corresponding distal cutaneous distribution of the injured peripheral nerve [38–40].

**Fig. 3:**
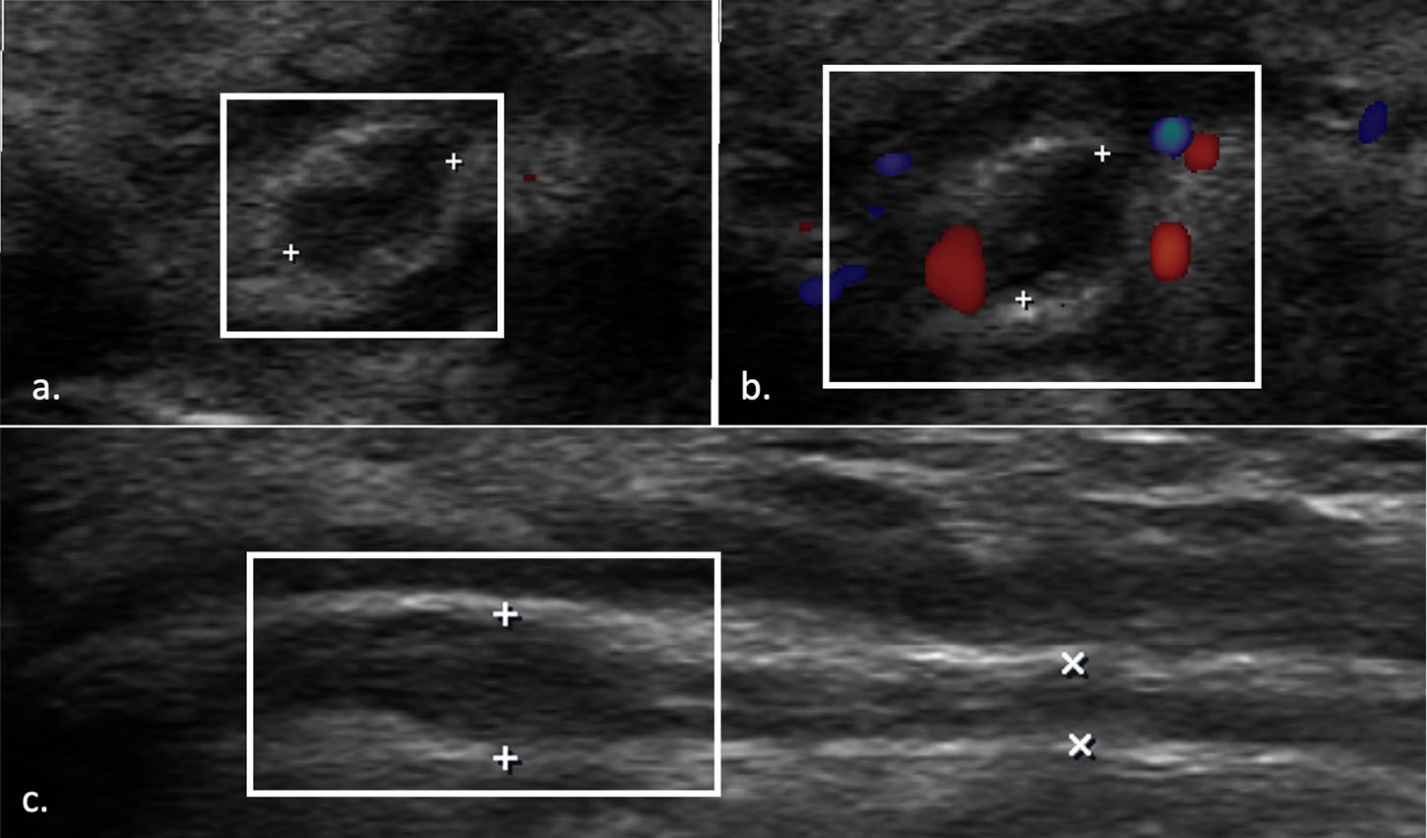
US of cubital tunnel syndrome. Transverse axis (**a**), color Doppler (**b**), and longitudinal axis (**c**) US images of UN within the cubital tunnel. The nerve is focally thickened with loss of fascicular architecture and hypoechoic appearance. There is increased perineurium vascularity on color Doppler. *Case courtesy of Dr. Luca De Flaviis, Milano, Italy*

**Fig. 4:**
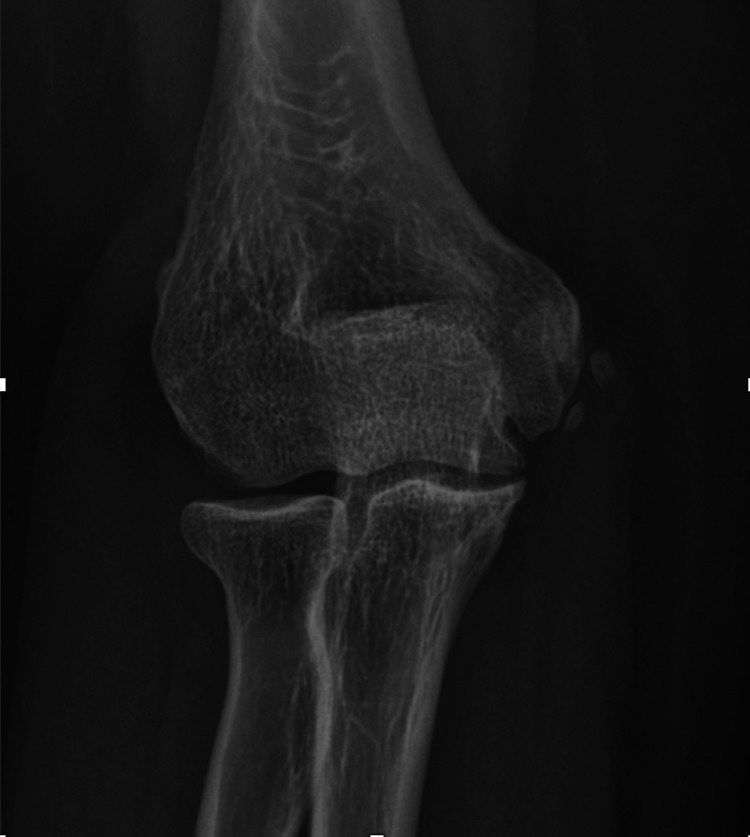
Elbow X-ray showing signs of degenerative disease with osteophyte formation, joint space reduction, and medial periarticular ossifications that compress the UN

**Fig. 5:**
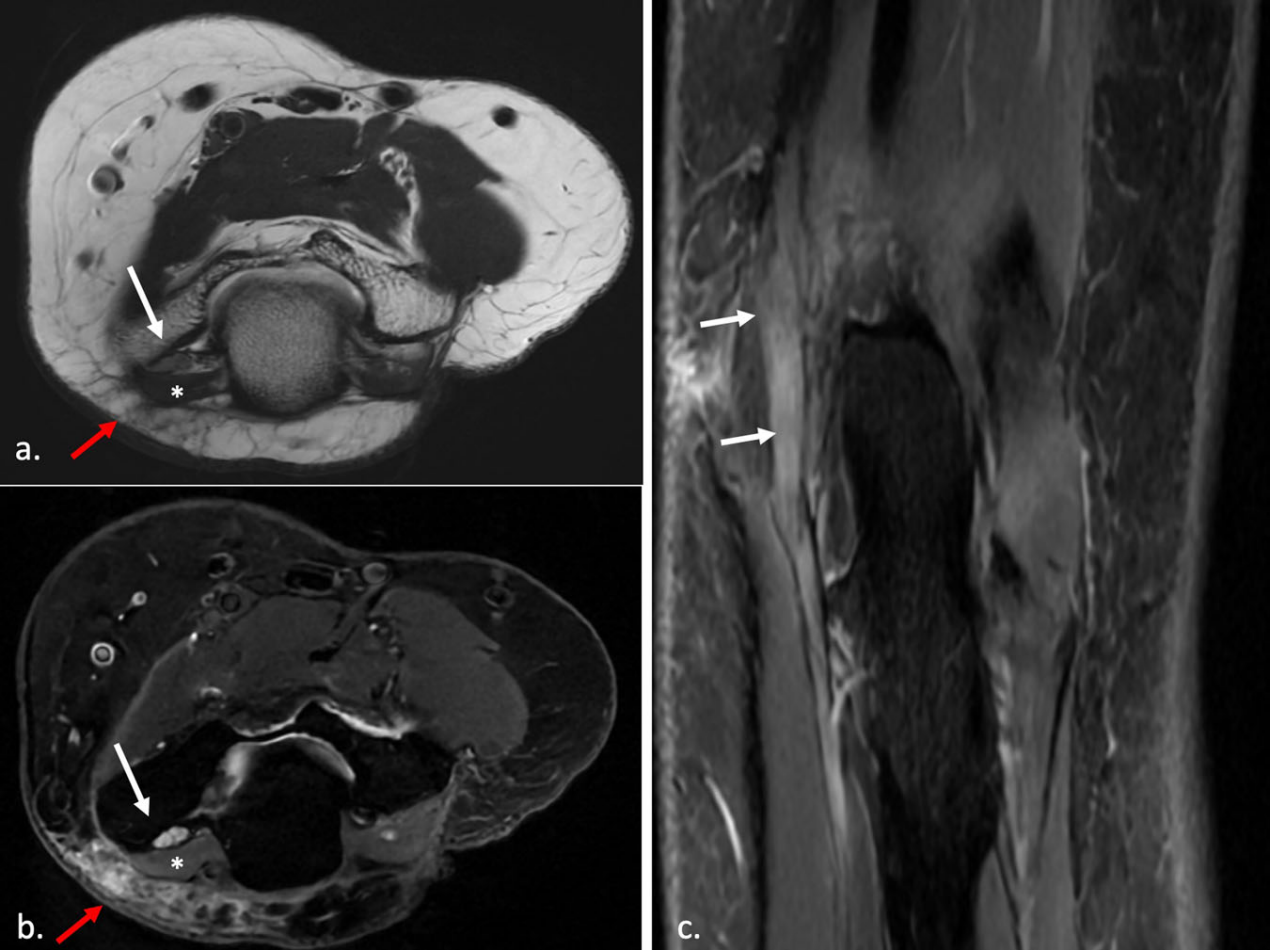
MRI of anconeus epitrochlearis muscle. Axial T1 (**a**), axial (**b**), and coronal (**c**) fat-suppressed PD images show an accessory anconeus epitrochlearis muscle (*), enlargement and hyperintensity of the UN in the cubital canal (white arrow) and edema of the surrounding soft tissues on the posteromedial aspect of the epitrochlea (red arrow)

US and MRI can show findings of neuropathy (thickening and loss of fascicular pattern) and/or of muscle denervation and concurrent causes of extrinsic compression, while plain radiographs can show fracture sites, soft tissue swellings, orthopedic implants, a bony lesion, or osteophytes in proximity to the course of the nerve [32] (Figs. 3–5).

### Ulnar nerve instability

The term “ulnar nerve instability” refers to chronic conditions where the UN partially or completely dislocates during elbow flexion and relocates during elbow extension [41]. UN dislocation can occur due to congenital partial or complete absence, laxity, or a tear of the retinaculum (Osborne ligament), a hypoplastic trochlea, posttraumatic cubitus valgus deformity, tight posterior medial collateral ligament bundle, or increased cubital tunnel pressure [42–45]. UN movement is not always associated with neuropathy, as several studies documented the presence of this condition in 2% to 49% of asymptomatic healthy elbows [46–49]. However, recurrent subluxation of the nerve at the elbow can cause tractional and frictional damage. Additionally, the nerve is more vulnerable to trauma in its partially dislocated position, where it lies superficially on the medial humeral epicondyle, potentially leading to ulnar neuropathy [41, 50]. When symptomatic, UN instability may present with pain in the medial elbow region, sometimes accompanied by tingling and paresthesia in the 4th and 5th fingers. Patients may report a “snapping” sensation and exhibit a positive Tinel sign. Clinical examination may reveal a palpable dislocation of the nerve over the medial epicondyle [46, 51].

### Snapping triceps syndrome

**Fig. 6:**
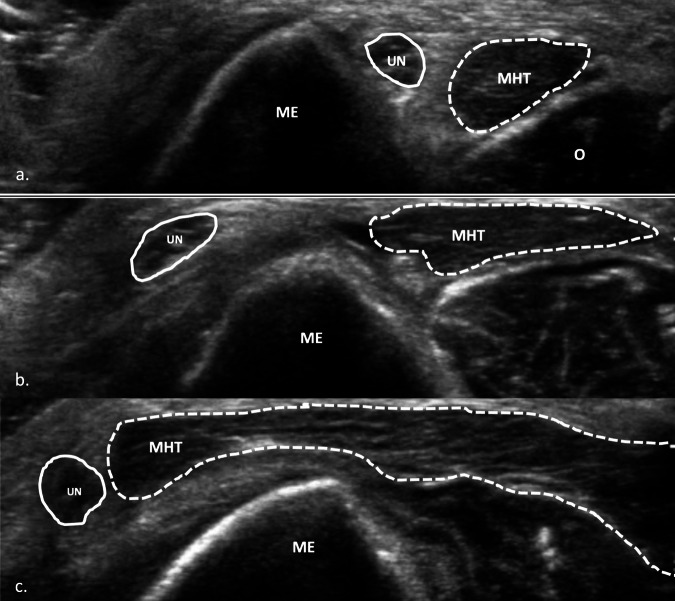
UN findings in dynamic US. **a** US image of UN in the transverse axis at the level of the cubital tunnel in elbow extension position (**a**), during partial elbow flexion (**b**), and complete elbow flexion (**c**). In **a**, the UN is located between the olecranon (O) and the medial epicondyle. In **b** the UN has crossed over the apex of the medial epicondyle to locate outside the cubital tunnel (1st snap). In **c**, both the UN and medial head of the triceps muscle have crossed beyond the apex of the medial epicondyle (2nd snap). ME, medial epicondyle; MHT, medial head of the triceps muscle

UN dislocation can be associated with snapping triceps syndrome, a condition where part of the triceps’ medial head dislocates along with the UN over the medial epicondyle. This occurs when the elbow moves from a fully extended position to full flexion or vice versa [52]. These dislocations result in at least two snaps at the elbow: the first snap is caused by the UN, followed by the medial head of the triceps muscle. Often, the snapping of the medial head of the triceps muscle over the medial epicondyle is subtle and detectable only with careful palpation, though it can sometimes be seen or heard. Dynamic ultrasound evaluation in three different positions (extension, 90-degree flexion, and full flexion) can demonstrate this condition and may also detect signs of ulnar neuropathy [29, 53] (Fig. 6). Differential diagnosis between simple UN instability and snapping triceps syndrome is crucial, as treatment of the two entities is different.

## Median nerve

The MN originates from the medial and lateral cords of the brachial plexus (C6–C8, T1) and travels anterior to the brachial artery in the anterior-medial compartment of the arm. Just above the elbow, it passes through the antecubital fossa, lying posterior to the biceps aponeurosis (also known as lacertus fibrosus) and anterior to the brachialis muscle. At the elbow joint, the nerve is situated between the humeral (superficial) and ulnar (deep) heads of the pronator teres muscle and enters the anterior compartment of the forearm by passing under the fibrous arch of the two heads of the flexor digitorum superficialis muscle. In the forearm, the nerve runs between the flexor digitorum superficialis and profundus muscles [54, 55]. At the elbow and in the proximal forearm, the MN innervates the pronator teres, flexor carpi radialis, palmaris longus, and flexor digitorum superficialis muscles. It also provides branches to the elbow and proximal radioulnar joints, a branch to the palmar skin, and muscular branches to the thenar eminence [56]. The anterior interosseous nerve (AIN), the largest branch of the MN, arises approximately 5 to 8 cm distal to the level of the lateral epicondyle. It follows the anterior aspect of the interosseous membrane with the anterior interosseous artery, running between the flexor pollicis longus and the flexor digitorum profundus muscles towards the wrist. The AIN is a purely motor nerve, supplying the pronator quadratus muscle, the radial half of the flexor digitorum profundus for the index and middle fingers, and the flexor pollicis longus muscle [57, 58]. The palmar cutaneous nerve arises from the MN about 7 cm proximal to the distal wrist crease and supplies the skin over the palm and the proximal aspect of the thenar eminence [59].

For US examination of the MN, the transducer is placed transversely on the anterior aspect of the elbow joint. At this level, the MN runs medial to the brachial artery and between the humeral and ulnar heads of the pronator teres muscle. Here, the brachial artery branches into the radial and ulnar arteries. The ulnar artery travels deep to the ulnar head of the pronator teres, and the MN can be observed in the proximal forearm along the medial aspect of the ulnar artery [36]. Videos 05-06 show how to scan the MN from the upper arm to the elbow (Video 05) and from the elbow to the forearm (Video 06).

### Kiloh-nevin or AIN syndrome

AIN syndrome is characterized by absent motor function in the muscles supplied by the AIN and is quite rare, comprising less than 1% of all nerve syndromes of the upper limb. The exact etiology and pathophysiology of AIN remain unclear, although common theories suggest either an idiopathic immune-mediated neuritis or intrinsic compression of the AIN around the elbow [60, 61]. Causes of AIN compression include the heads of the pronator teres muscle, the proximal edge of the flexor digitorum superficialis arch, enlarged bicipital tendon bursa, lacertus fibrosus, a thrombosed radial artery branch in the mid-forearm, a thrombosed ulnar artery, tendinous bands, and osseous spurs. Additionally, the AIN can be entrapped by anatomical variants such as Gantzer’s muscle, which is an anomalous head of the flexor pollicis longus [62]. Direct traumatic injury to the AIN from trauma (e.g., penetrating injuries, forearm fractures, venipuncture), extrinsic masses (e.g., tumors), or external compression of the forearm (e.g., from guitar playing, shoulder immobilization slings, crutches) is rare and not considered true AIN syndrome due to differing pathophysiology, with symptoms often resolving once the external compression is eliminated [63–65].

Patients with AIN syndrome typically present with pain in the forearm along the course of the nerve and weakness in the flexor pollicis longus and flexor digitorum profundus muscles, often complaining of an inability to button shirts or turn car keys. Because the AIN does not provide sensory innervation to the skin, sensory deficits do not occur with isolated AIN palsy [66].

Physical examination may reveal the Kiloh-Nevin sign, where patients cannot make an “OK” sign with the thumb and index finger. Additionally, the Pinch Grip test will be positive: in patients with AIN syndrome, the thumb interphalangeal joint and the distal interphalangeal joint of the index finger remain extended while attempting to pinch a sheet of paper [67].

**Fig. 7:**
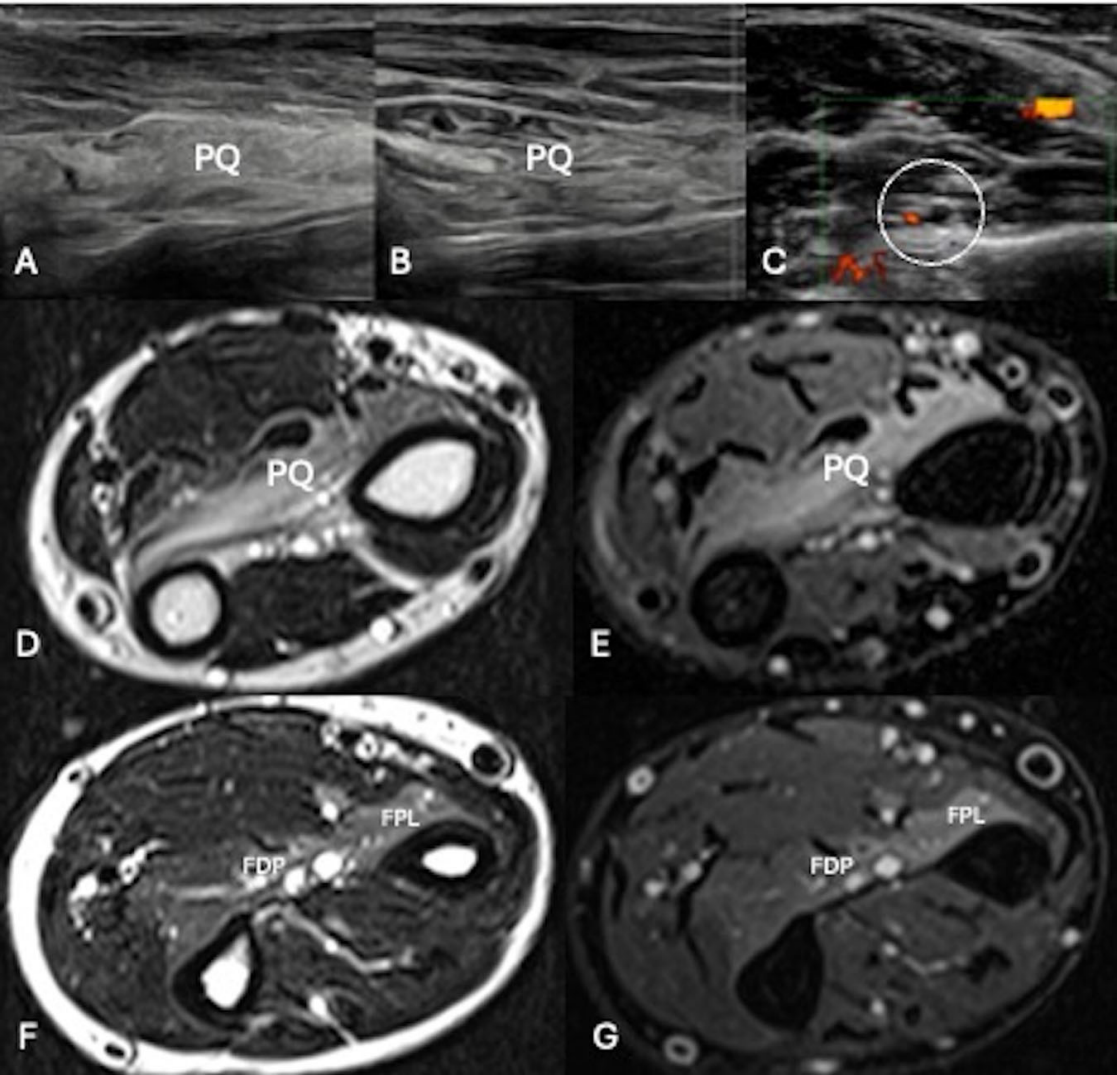
US and MRI images AIN syndrome. US images of denervation (**A**) of the pronator quadratus muscle, which appears thinner and diffusely hyperechoic, versus US normal appearance of the muscle in the unaffected side (**B**). Axial image of the volar aspect of the mid-third of the forearm (**C**) shows the thickening of the AIN running close to the anterior interosseous artery (circle). Axial T2-weighted (**D**, **F**) and axial fat-suppressed PD (**E**, **G**) MRI images show edema of pronator quadratus muscle (PQ), the radial half of the flexor digitorum profundus (FDP), and the flexor pollicis longus (FPL) due to denervation

Diagnosis is typically based on clinical signs and electrodiagnostic examination. US visualization of the AIN is challenging due to its small diameter but may help identify the cause of extrinsic compression, assess signs of muscular denervation, and exclude possible alternative diagnoses such as lesions of the flexor digitorum profundus and flexor pollicis longus tendons. In this case, MRI may be more helpful than the US, as the visualization of involved muscles may represent the only imaging sign of this pathology. This is also clinically significant for assessing the chronicity of denervation and the probability of successful surgical repair [68] (Fig. 7).

### Supracondylar process syndrome

**Fig. 8:**
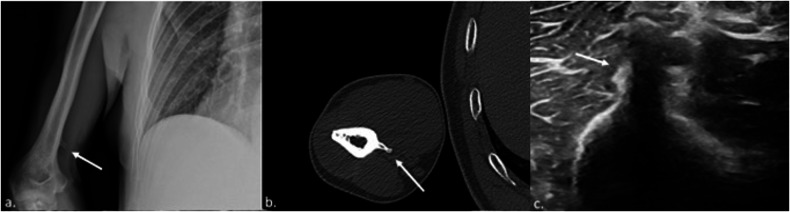
X-ray (**a**), CT (**b**), and US (**c**) of the supracondylar process (arrow)

A supracondylar process is a bony spur arising from the anteromedial humerus, approximately 3–6 cm proximal to the medial epicondyle, and is present in 1–2% of individuals. The ligament of Struthers is a fibrous remnant of a tendinous structure extending from the tip of the process to the medial epicondyle. The MN and brachial artery usually pass under this ligament. The process can exist without an associated ligament, and the ligament can be present without the process, attaching directly to the humeral shaft. Supracondylar process syndrome refers to the compression of the MN under this bony process or ligament [69–71]. Most patients exhibit symptoms of MN compression, such as tingling, numbness, and weakness, with pain often worsening during forearm extension and pronation. Common signs include tenderness medially above the elbow and a palpable process [72]. The supracondylar process can be easily detected on X-ray images obtained in oblique views but may not be identified in anteroposterior or lateral views alone [73]. On US evaluation, the supracondylar process appears as a thick echogenic line with distal acoustic shadowing, while the Struthers’ ligament appears as a thin, hypoechoic linear structure [74] (Fig. 8). On MRI, Struthers’ ligament can be visualized in both T1 and T2-weighted images as a thin, linear, hypointense structure running from the tip of the supracondylar process to the medial epicondyle. The MN and UN, along with the brachial artery, can be found running underneath the ligament [75].

### Pronator teres syndrome

**Fig. 9:**
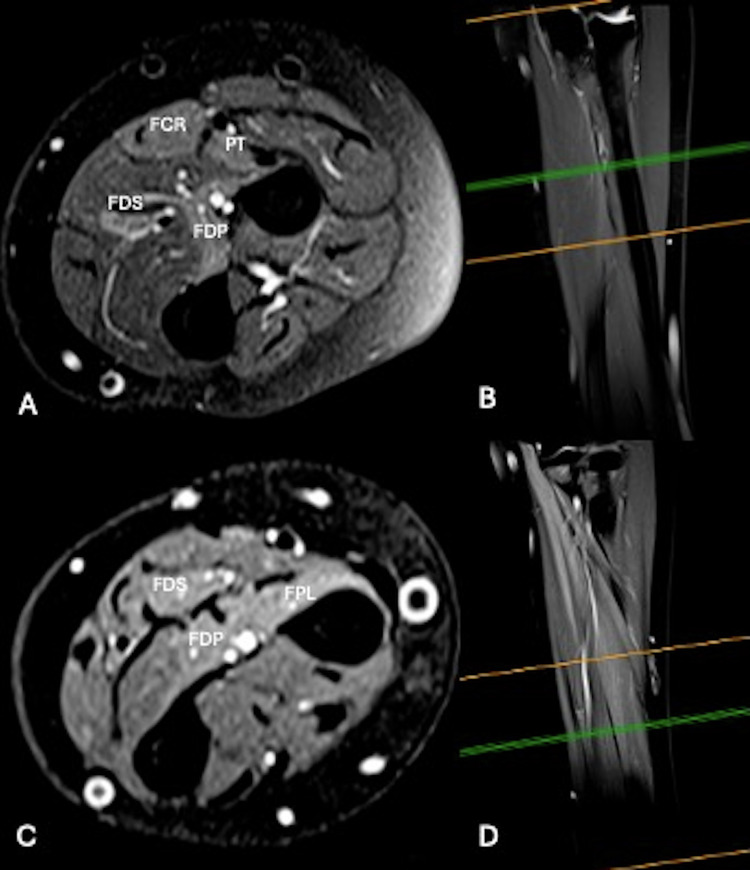
Pronator teres syndrome. Axial fat-suppressed PD MRI images (**A**, **C**) at the proximal (reference in **B**) and distal (reference in **D**) third of the forearm show edema of pronator teres (PT), flexor carpi radialis (FCR), flexor digitorum superficialis (FDS), radial half of the flexor digitorum profundus (FDP) and flexor pollicis longus (FPL) muscles due to denervation

Pronator teres syndrome refers to the compression of the MN as it passes between the ulnar and humeral heads of the pronator teres muscle. This condition is typically caused by muscle hypertrophy due to excessive overloading and repetitive grasping or pronation movements, such as prolonged hammering, ladling food, cleaning dishes, or playing tennis. It can also result from local trauma leading to hematoma or deformity, the presence of masses like Schwannomas or lipomas, post-puncture hematomas in patients undergoing anticoagulation therapy, or a dialysis fistula [76, 77]. The risk of MN compression increases with certain anatomical variations, such as when the ulnar veins pass through the pronator canal with the MN, when the MN runs through the ulnar head of the pronator teres, or when the ulnar head is fibrous [78]. Pronator teres syndrome is a rare condition that may be easily overlooked and mistaken for the more prevalent carpal tunnel syndrome due to overlapping symptoms [69]. Patients with pronator teres syndrome may present with numbness or paresthesia in the radial three-and-a-half digits, as well as pain and tenderness over the pronator muscle, often associated with a positive Tinel’s sign over the MN at that point. Unlike carpal tunnel syndrome, intense nocturnal paraesthetic discomfort is uncommon. On physical examination, a positive pronator compression test is the most common sign of pronator syndrome. This test is performed by applying pressure proximal and lateral to the proximal edge of the pronator teres muscle belly on the volar forearm, which produces pain or paresthesia within 30 s of compression [67, 79]. In the US, the pronator teres muscle can appear thicker and hyperechoic compared to the contralateral side. The US can also highlight the presence of intramuscular masses or hematoma, and the MN may appear flattened between the two heads of the pronator teres. Dynamic US evaluation is performed with the palm of the patient’s hand in that of the examiner, allowing sonographic assessment during resisted pronation. This can show adhesion of the MN in the pronator tunnel, resulting in synchronized movements with the pronator teres muscle [80, 81]. MRI can show edema and/or atrophy of pronator teres, flexor carpi radialis, palmaris longus, and flexor digitorum superficialis muscle bellies, in addition to those muscles innervated by the AIN (Fig. 9).

## Radial nerve

The RN is the terminal branch of the posterior cord of the brachial plexus (C5–C8, T1). In the arm, it travels posterior to the humerus in the spiral groove, then penetrates the lateral intermuscular septum to exit the groove and lies anterior to the lateral condyle beneath the brachioradialis muscle [82]. At the elbow joint, the RN divides into a superficial sensory branch, which runs in the forearm between the brachioradialis and supinator muscles, and a deep motor branch, known as the posterior interosseous nerve (PIN), which passes beneath the arcade of Fröhse—a fibrous arch formed by the proximal thickened edge of the superficial head of the supinator muscle in 35% of individuals. The nerve then travels between the superficial and deep heads of the supinator muscle and exits the distal edge of the supinator muscle into the posterior compartment of the forearm [83].

Proximally, the RN innervates the medial and long heads of the triceps and the anconeus muscles. In the distal arm, it provides motor branches to the brachialis, brachioradialis, and extensor carpi radialis longus and brevis muscles, as well as supplying the elbow joint and the skin on the radial aspect of the proximal forearm. The PIN innervates the supinator muscle as it passes between its two heads, and distally provides branches to the extensor muscles of the wrist and hand, including the extensor digitorum communis, extensor digiti minimi, extensor carpi ulnaris, extensor pollicis longus and brevis, abductor pollicis longus, and extensor indicis proprius muscles. The superficial RN innervates the skin of the thumb, index, and middle fingers [84, 85].

For the US examination of the RN, the transducer is placed transversely on the anterolateral elbow, with the patient seated in front of the examiner with the arm in a neutral position. The examination follows the main trunk of the nerve as it courses adjacent to the bone between the brachioradialis and brachialis muscles until its bifurcation into the superficial sensory branch and the PIN. In normal subjects, the measured diameters of the nerve in this area are 4.0–4.2 mm (latero-lateral) and 2.3–3.5 mm (anteroposterior). Evaluating the PIN is facilitated by positioning the elbow in semiflexion over the examination table with the forearm oriented transversely and sweeping the probe over the supinator during hand extension and pronation; the nerve may appear to follow an angulated course at the arcade of Fröhse [86, 87]. Videos 07–09 show how to scan the RN from the upper arm to the elbow, the PIN (Video 08), and the superficial sensory branch of the RN (Video 09). RN neuropathies occur less frequently than those of the MN and UN because the RN is less commonly involved in entrapment syndromes. However, it is more often damaged by arm trauma, usually secondary to fractures [88]. Predisposing factors for nerve compression include overuse, particularly repetitive supination and pronation motions, high-intensity handgrip force, and prolonged exposure of the arms to vibration, such as from excessive typing (occupational) and playing tennis (recreational) [89].

### Posterior interosseous nerve (PIN) syndrome

**Fig. 10:**
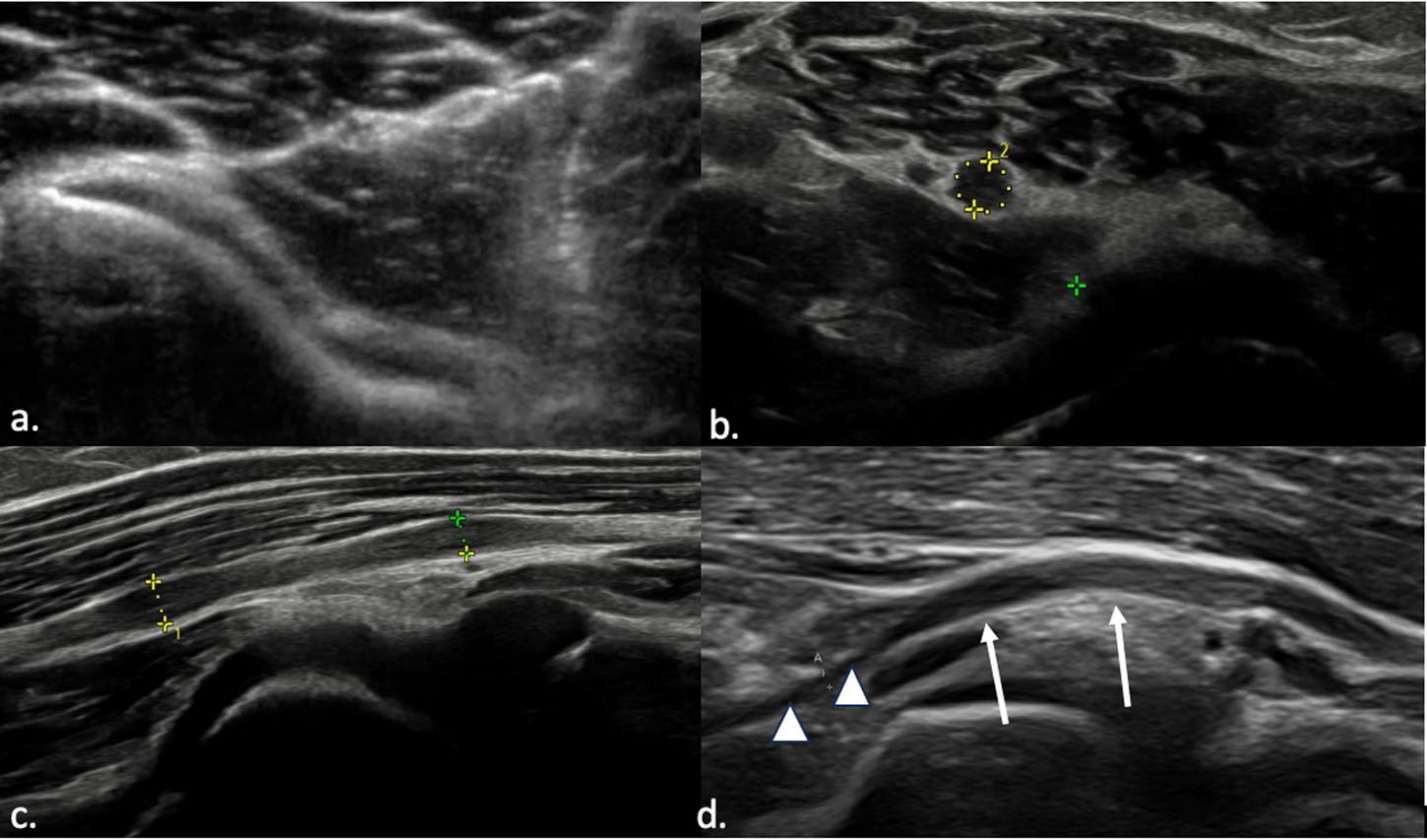
US image of posterior interosseous nerve. Normal appearance (**a**) and pathologically enlarged and hypoechoic nerve (**b**) as it passes through the Fröhse arcade. Long axis images (**c**, **d**) show diffuse swelling of the nerve and proximal nerve enlargement (straight arrows) compared to normal nerve size (arrowheads) as the nerve enters the supinator muscle (**d**)

PIN syndrome is the most common compressive neuropathy affecting the RN. There are five primary sites of PIN entrapment, with the most frequent being the arcade of Fröhse (Fig. 10). Video 10 shows a case of entrapment of the PIN within the arcade of Fröhse. Other potential entrapment points include: the medial margin of the extensor carpi radialis brevis and the aponeurosis located beneath it, blending with the deep fascia covering the flexors; the distal border of the supinator muscle; the floor of the radial tunnel, which consists of fibrous tissue arising from the radial head and fusing with the brachialis muscle, extensor carpi radialis brevis, and the superficial head of the supinator; another possible compression site is at the level of the radial neck, where hypertrophy of the recurrent radial vessels (known as the leash of Henry) can induce compression. Additionally, ganglion cysts, lipomas, synovial pathologies, radial head fractures, and Monteggia fracture dislocations can also cause compression of the PIN [85, 90]. Clinical findings of PIN syndrome include pain in the lateral aspect of the forearm and hand, weakness in finger extension, and atrophy of the forearm muscles, all without sensory deficits. This distinguishes it from radial tunnel syndrome, which typically causes pain without motor deficits [91]. US and MRI can reveal signs of neuropathy and distal muscle denervation, specifically affecting only the distal common extensor muscle group (extensor carpi radialis brevis, extensor digitorum, and extensor carpi ulnaris) and the supinator muscle [92].

### Spiral groove syndrome

The RN is especially susceptible to entrapment at the level of the spiral groove of the humerus because of its proximity to the bone cortex [93]. The RN is often injured by trauma, typically due to spiral fractures between the middle and distal thirds of the humeral shaft with fragment displacement. It can also be injured during fracture manipulation or reduction, or because of metal fixation devices such as screws and plates. Other potential causes of RN compression in the arm include tumor growth, prolonged inflation of an automatic blood pressure cuff around the arm, or the presence of an anomalous brachioradialis muscle. Less commonly, radial neuropathy can result from external nerve compression in the spiral groove, a condition known as “Saturday night palsy” [94]. The term “Saturday night palsy” originates from its association with spending a night in an alcoholic stupor with the arm draped over a chair or bench. Mechanical compression of the RN in the spiral groove can also result from the continuous use of crutches or prolonged kneeling in a shooting position [93]. When the RN is injured in the spiral groove or mid-arm, patients typically experience wrist drop and loss of sensation over the lateral aspect of the arm and forearm, the thenar eminence, and the radial three-and-a-half digits [95]. US and MRI can be used to follow the RN at the site of compression at the level of the spiral groove, as well as to check the integrity of the nerve and to identify extensor muscle denervation in the forearm. Plain radiography and CT are helpful for detecting bone fractures and the position of metal devices. Video 11 shows a case of spiral groove syndrome.

## Conclusions

Imaging plays just a role in the correct interpretation of elbow neuropathies, which relies on the knowledge of the course, function, and pathology of peripheral nerves, the awareness of sensory and motor innervation, and the subsequent clinical picture associated with entrapment neuropathies. US is certainly superior to MRI for evaluating nerves’ structure and dynamic motion, but it is less sensitive to muscle changes associated with denervation. The knowledge of anatomical landmarks (vessels, muscles, tendons, bones) and sites of compression is essential for being used as guidance for the assessment of peripheral nerves.

## Supplementary information

Video 1

Video 2

Video 3

Video 4

Video 5

Video 6

Video 7

Video 8

Video 9

Video 10

Video 11

## Abbreviations

AIN: Anterior interosseous nerve
CSA: Cross-sectional area
MN: Median nerve
PIN: Posterior interosseous nerve
RN: Radial nerve
UN: Ulnar nerve
